# The Relationship between CPAP Usage and Corneal Thickness

**DOI:** 10.1371/journal.pone.0087274

**Published:** 2014-01-24

**Authors:** Ethem Gelir, Murat Timur Budak, Sadik Ardıc

**Affiliations:** 1 Physiology Department, Hacettepe University Medical School, Ankara, Turkey; 2 Sleep Laboratory, Pulmonary Medicine Department, SGK Ankara Education Hospital, Ankara, Turkey; University of Alabama at Birmingham, United States of America

## Abstract

The purpose of this study was to determine whether there is a correlation between CPAP usage and corneal thickness in patients with sleep disordered breathing. Full-night polysomnography (PSG) recordings were collected. Ten patients had undergone PSG recordings with continuous positive airway pressure (CPAP), and seven patients had undergone PSG recordings without CPAP. We measured corneal thickness by ultrasonic pachymeter before sleep and ten minutes after waking. We also measured visual acuity with a routine ophthalmologic eye chart before and after sleep. We asked patients to fill out a post-sleep questionnaire to get their subjective opinions. In the without-CPAP group, corneal thickness increased significantly during sleep in both eyes (left, p = 0.0025; right, p<0.0001). In the with-CPAP group, corneal thickness did not increase significantly (p>0.05 for both left and right cornea). There was no significant difference in visual acuity tests (p>0.05 for both left and right eye) between the two groups. According to our results, there is a significant increase in corneal thickness in the without-CPAP group. Our data show that a low percentage of Rapid Eye Movement (REM) sleep may cause an increase in corneal thickness, which can indicate poor corneal oxygenation. In fact, many sleep-disordered breathing (SDB) patients have low REM. Since a contact lens may cause low corneal oxygenation, SDB patients with contact lenses should be monitored carefully for their corneal thickness.

## Introduction

SDB is the most common sleep disorder associated with excessive sleepiness. SDB is characterized by episodes of sleep apnea (cessation of breathing over 10 s or more) or hypopnea (significant reduction of breathing), oxygen desaturations, and frequent arousals [1]. The most common form of SDB is obstructive sleep apnea (OSA) and is associated with airway collapse as the cause of breathing cessation or reduction. The standard, first-line treatment for OSA is continuous positive airway pressure (CPAP) [2]. CPAP is fan-generated air pressure delivered via a nasal mask and titrated to offset negative intrathoracic pressures produced during inhalation. As such, CPAP acts as a pneumatic splint to maintain airway patency. It has been shown to be very effective in most patients leading to improved daytime alertness, cognitive function, and quality of life [3]–[5].

Since the discovery of rapid eye movement (REM) sleep in 1953, it has been established that REM sleep is homeostatically regulated. Selective REM sleep deprivation produces compensatory increases in REM on subsequent sleep opportunities [6]. This phenomenon is commonly called “REM rebound”. REM rebound occurs regardless of whether the original REM suppression was instrumental [7] (i.e., waking subjects up when they entered REM sleep), pharmacologic [8] (e.g., amitryptaline or fluoxetine), or disease related [9] (e.g., sleep-related breathing disorders). Subsequently, researchers have found that in adults, REM occupies 20–25% of total sleep time and many physiologic changes are associated with REM sleep, including atonia [10], poikilothermia [11], nocturnal penile tumescence [12], [13], middle ear muscle activity [14], and increased cerebral blood flow [15].

Recently, corneal thickening was added to the list of physiologic properties affected by REM sleep. The hypothesis was advanced by Maurice (1998) who proposed that eye movements in REM sleep help corneal oxygenation. According to Maurice, thermal circulation of the aqueous humor is needed for adequate corneal respiration. This circulation is suppressed when the lids are closed, and REM is required to stir the anterior chamber and thus prevent corneal anoxia during sleep [16].

Corneal thickness measurements give valuable information about the physiological status of the cornea [17]–[19]. Healthy human corneal thickness is around 500 microns [20]. However the thickness can change under some circumstances, such as hypoxia and hypercapnia. It has also been shown that corneal thickness significantly affects intraocular pressure measurement, and may itself be a risk factor for developing glaucoma [21], [22]. Early studies showed that the normal human cornea would swell by 7% every hour in an oxygen-free environment [23]. Diurnal variation of central corneal thickness (CCT) has also been described, with swelling overnight; this swelling resolved by early afternoon, suggesting it was caused by the lid closure creating hypoxia [24]. Corneal swelling caused by hypoxia is a well-known phenomenon, especially in relation to contact lens wear [25]. Long-term use of contact lenses was shown to alter the following conditions in the cornea: epithelial oxygen uptake, epithelial thickness, stromal thickness, and corneal endothelial morphology [26], [27].

OSA causes decreased REM sleep percentage and CPAP usage can usually reverse this decrease. If Maurice’s hypothesis is right, then decreased REM percentage should jeopardize corneal oxygenation. So far, no study has elucidated the relationship between the CPAP usage and the corneal thickness. Thus, the purpose of this study was to determine whether a correlation exists between CPAP use and corneal thickness in patients with OSA.

## Materials and Methods

### Ethics statement

We obtained Institutional Review Board approval from Baylor College of Medicine (Houston,TX) for the procedures of the study. Our study has been carried out in accordance with The Code of Ethics of the World Medical Association (Declaration of Helsinki) for experiments involving humans.

### Subjects

In this study, patients underwent standardized sleep center clinical procedures. Men and women, admitted to the Sleep Laboratory at Baylor College of Medicine, Michael E. DeBakey Veterans Affairs Medical Center, for overnight polysomnography, were eligible for this study. Subjects who met the following inclusion criteria were selected for this study: patients must be diagnosed with OSA, be 21–65 years of age (inclusive), and provide written informed consent. We excluded patients with eye (e.g., glaucoma) or neurological (e.g., periodic leg movement) diseases. Twenty patients participated in the study, subjects were randomly assigned to one of two groups (10 subjects in each group). Patients who had used CPAP were called the “with-CPAP” group, and patients who had not used CPAP were called the “without-CPAP” group. However three subjects in the “with-CPAP” group were excluded because they did not tolerate the CPAP treatment. All subjects (1 woman and 16 men) were CPAP naïve. The “with-CPAP” group subjects underwent full-night manual CPAP titration. The mean age in the with-CPAP group was 57±3.5 years and in the without-CPAP group 59±2.2 years (mean ± S.E.M.). These age distributions were normal, as demonstrated by normograms. The only additional procedures for this study were pre- and post-sleep ultrasonic corneal thickness measurement and visual acuity testing.

### Corneal thickness measurement

A DGH500 Pachette ultrasonic pachymeter with a hand-held transducer (DGH Technology, Inc. Exton,PA) was used to measure corneal thickness. The ultrasonic pachymeter calculates readings based on an ultrasonic velocity of 1640 m/s for normal human corneal thickness. To locate the center of the cornea, each subject was instructed to stare directly at an ophthalmic pen light positioned at eye level, approximately 2 meters from the subject. Although there are other methods to measure corneal thickness, such as optic and electronic digital methods, the ultrasonic method is the most preferred one, because it is accurate, easy to use, and reliable [28], [29]. Thickness measurement of each cornea was done 15 times for each measurement and the arithmetic mean of 15 measurements was used as a parameter of statistical analysis of each eye.

### Visual acuity testing

This procedure involves a standard eye chart. A second chart was used to prevent memorization of letters by the subject, as each individual was exposed to the chart twice. Right and left eyes of patients were examined separately to assess visual acuity. In this method, normal vision was accepted as 20/20 (Snellen unit). We transformed these values to their decimal equivalents as 20/20  =  1. Normal vision is represented by “1”. So, an increase in this numerical value should be considered as better vision and vice versa. These decimal values were then converted into a logarithm of the minimum angle of resolution (logMar) equivalents.

### Standardized sleep center clinical procedure

Analysis included overnight polysomnography for diagnosis followed by a night of CPAP titration. These procedures were performed as part of a routine clinical evaluation. Full-night PSGs were recorded according to standard practice. We made sleep recordings using Grass Heritage computerized polysomnographic systems. Standard surface electrodes were used to record electroencephalographic, electrooculographic, electromyographic (submental and anterior tibialis), and electrocardiographic activities. Nasal oral thermocouples monitor airflow, while thoracic and abdominal movements indicate respiratory effort. Respiratory tracings were scored for the presence of apneas (a 10 second, or longer, cessation in nasal-oral airflow) or hypopneas (a 10 second, or longer, reduction of nasal-oral airflow of 50%, or more). Blood oxygen saturation was monitored with pulse oximetry (with the sensor placed on the earlobe). Sleep latency, sleep efficiency, percentage of time in each sleep stage, and other polysomnographic parameters were calculated. Recording and scoring techniques followed currently published standards for human subjects; this includes procedures for sleep stages [30], respiration [31], and leg movement [32]. In the morning, monitoring devices were removed and the subject completed a post-sleep questionnaire detailing subjective sleep quality and quantity. A registered polysomnographic technologist analyzed the sleep record and oximetry data. Then, a staff physician reviewed the records. In addition to the mentioned measurements, we also elicited REM density value, which measures the frequency of rapid eye movements during REM sleep. The formula used to calculate REM density is described elsewhere [33].

### Sleep questionnaires

As a part of routine sleep center clinical procedure, we administered a post-sleep questionnaire to subjects. In this questionnaire, among many others, there were four questions regarding vision after sleep. These questions were about “Clearer Vision”, “Dimmer Vision”, “Blurred Vision”, and “Eye Discomfort”. They were asked to check one of the three options, which were “less”, “same”, and “more”.

## Results

### Subjects

There was no statistically significant difference in the age distribution between the two groups (p =  0.35). Thus, the groups were considered comparable. In this study, patients underwent standardized sleep center clinical procedures.

### Corneal thickness versus REM percentages and REM density

Statistical analysis for corneal thickness was performed by Student’s t-test using GraphPad InStat 3.1 and Prism 5.03, GraphPad Software, San Diego, CA, USA. In the without-CPAP group, corneal thickness increased significantly after one night’s sleep. The two-tailed p value was 0.0025 for left cornea, and p was < 0.0001 for right cornea. In the with-CPAP group, corneal thickness did not increase significantly after one night’s sleep, as measured using a two-tailed t-test.

The average percentages of REM sleep were 13.0±2.1 and 15.98±4.0 in without-CPAP and with-CPAP groups, respectively. An Unpaired test was performed to compare REM sleep percentages of both groups and no significant difference was found. The Pearson correlation coefficient was calculated to test the null hypothesis that there was no correlation between REM sleep percentages and corneal thickness values. No significant correlation was found (r^2^ = 0.124 for right cornea, 0.187 for left cornea in with-CPAP group and; 0.123 for right cornea, 0.275 for left cornea in without-CPAP group). Corneal thickness values of both groups are listed in Table 1, and associated REM sleep percentages and REM density values are listed in Table 2. Values are given as microns for corneal thickness, and as a percentage for REM sleep (mean ± S.E.M.).

**Table 1:** Pre and post sleep comparison of left and right corneal thickness of each group.

Group	n	Pre-Sleep Left Cornea	Post-Sleep Left Cornea	Pre-Sleep Right Cornea	Post-Sleep Right Cornea
**Without-CPAP**	10	537.5±11.6 *	553.5±13.2	538.0±10.8 **	559.0±11.5
**With-CPAP**	7	544.8±9.5	545.2±10.2	545.7±8.9	551.4±9.7

Values are given as microns for corneal thickness (means ± S.E.M.).

* p = 0.0025 very significant (t = 4.134 with 9 degrees of freedom). The comparison is between Pre-Sleep and Post-Sleep of left corneal thickness in without-CPAP group.

** p< 0.0001 extremely significant (t = 9.257 with 9 degrees of freedom). The comparison is between Pre-Sleep and Post-Sleep of right corneal thickness in without-CPAP group.

**Table 2:** Demographic and polysomnographic variables in all subjects.

Group	n	Age (years old)	BMI (kg/m^2^)	SpO_2_ (%)	Pre-CPAP AHI	AHI	Baseline REM (%)	REM (%)	REM Density
**Without-CPAP**	10	59±2.2	30.4±1.8	90.9±1.2	---	24.0±3.2	---	13,0±2,17	9.1±0.6
**With-CPAP**	7	57±3.5	32.5±2.1	92.6±1.9	25.9±3.1	6.5±0.7	12,3±2,0	15,9±4,0	11.5±0.7
**p value**	---	0,6730	0.4883	0.4615	---	0.0005	---	0.4858	0.0354

All values are presented as means ± S.E.M.

The average REM density values were 9.1±0.6 and 11.5±0.7 in without-CPAP and with-CPAP groups, respectively. An Unpaired test was performed to compare REM density values of both groups and, we observed that the REM density value of the with-CPAP group is noticeably higher than that of the without-CPAP group (p< 0.05). The Pearson correlation coefficient was calculated to test the null hypothesis that there was no correlation between REM density and corneal thickness values. No significant correlation was found (r^2^ = 0.308 for right cornea, 0.458 for left cornea in with-CPAP group and; 0.003 for right cornea, 0.015 for left cornea in without-CPAP group).

### Visual acuity

**Figure 1:**
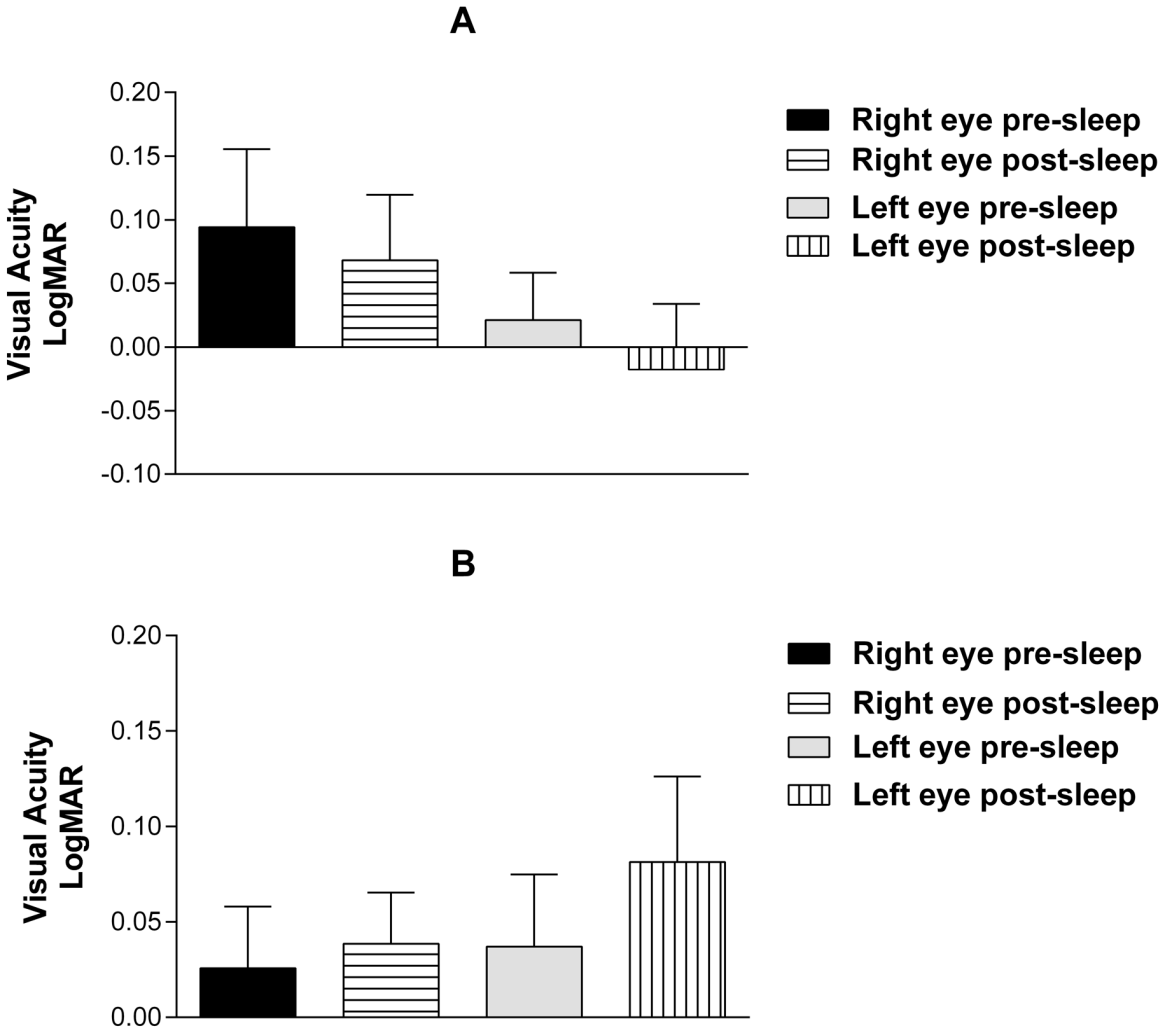
Results of eye chart visual acuity test for the without-CPAP group (A) and with-CPAP group (B) are presented in bar graph. There is no significant difference between groups. Error bars  =  S.E.M.

Eye chart examination revealed vision improvement in 7 eyes and deterioration in 3 eyes, while it revealed no change in 10 eyes of the without-CPAP group (10 patients, 10 pairs of eyes). In the with-CPAP group (7 patients, 7 pairs of eyes), vision improvement was observed in only one eye, and deterioration was observed in 5 eyes, while no change was observed in 8 eyes. There was no statistically significant difference in visual acuity before and after sleep (p>0.05) for both groups. Visual acuity values of each patient before and after sleep are given as Snellen units in Table 3. Figure 1 shows eye chart visual acuity results as logMar equivalents.

**Table 3:** Visual acuity values of each patient.

Without-CPAP Group				With-CPAP Group			
Right Eye Pre-sleep	Right Eye Post-sleep	Left Eye Pre-sleep	Left Eye Post-sleep	Right Eye Pre-sleep	Right Eye Post-sleep	Left Eye Pre-sleep	Left Eye Post-sleep
20/16	20/16	20/16	20/16	20/16	20/16	20/16	20/16
20/20	20/20	20/16	20/16	20/25	20/20	20/25	20/25
20/60	20/40	20/20	30/20	20/25	20/25	20/30	20/40
20/20	20/16	20/20	20/16	20/20	20/25	20/20	20/25
20/16	20/20	20/16	20/20	20/25	20/25	20/25	20/25
20/20	20/20	20/25	20/25	20/25	20/25	20/25	20/25
20/20	20/20	20/20	20/20	20/16	20/20	20/16	20/20
20/25	20/20	20/20	20/16	---	---	---	---
20/30	20/30	20/40	20/25	---	---	---	---
20/50	20/50	20/25	20/30	---	---	---	---

Values are given as Snellen units.

### Overnight polysomnography values

Apnea-hypopnea index (AHI), blood oxygen saturation levels (SpO_2_) and REM Density values of both groups are given in Table 2. Values are presented as mean ± S.E.M. by using Student’s t test.

### Sleep questionnaires

**Figure 2:**
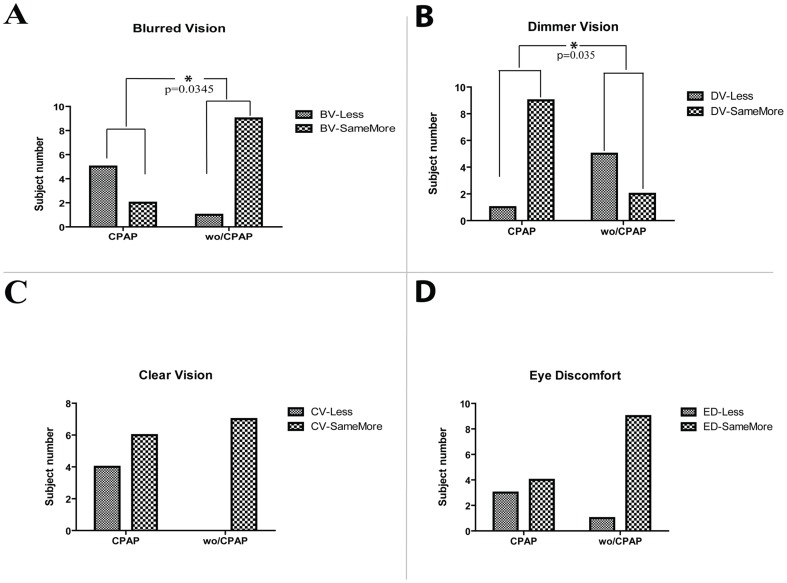
Results of post-sleep questionnaire of “with-CPAP” (CPAP) and “without-CPAP” (wo/CPAP) groups. The questionnaire is made-up of four categories, which are Blurred Vision (BV) – panel A, Dimmer Vision (DV) – panel B, Clear Vision (CV) – panel C and Eye Discomfort (ED) –panel D. Statistically significant (i.e., accepted as p<0.05) p values are shown in the figure. Non-significant p values are not shown. The possible three answers to each of the categories are summarized as “Less answer” =  “Less”, “Same answer”  =  “SameMore” and “More answer”  =  “SameMore” to make an easy interpretation of data.

According to the sleep questionnaire results, all of the with-CPAP group patients claimed that they had better vision after sleep. Our results showed that there were statistically significant changes in blurred vision and dimmer vision while there were no significant changes in clear vision and eye discomfort between groups. However, following Bonferroni adjustment for four comparisons and the adoption of p = 0.0125 as the border level of statistical significance, the indicated differences became statistically insignificant. Figure 2 shows these post-sleep questionnaire results.

## Discussion

In this study, we assessed the relationship between CPAP usage and corneal thickness. According to our results, there is a significant increase in corneal thickness in the without-CPAP group. Our results show that REM density value for the with-CPAP group was higher than that of the without-CPAP group (p< 0.05); however, the raw REM percentages of both groups were not significantly different, and no significant correlation was found between REM sleep percentages and corneal thickness values. The reason for this inconsistency is that REM sleep percentages and REM densities are different measurements, even though REM density is a derivative of REM percentage. REM density is defined as the percentage of time (in minutes) in which rapid eye movements occurred during the respective period of REM sleep, considering that amplitude of eye movements is higher than 25 mV [33]. Calculation of this index was made by the following equation: total minutes of rapid eye movements/total minutes of REM sleep x 100.

The tonic components of REM sleep (REM duration, the REM percent of sleep period time) may be increased while the REM density is significantly decreased due to prolongation of total REM duration and a parallel reduction of the total number of eye movements [34]. Several studies confirm the independence of REM density from other REM sleep parameters observed after selective paradoxical sleep deprivation and after the administration of cholinergic drugs [35]. Another reason for inconsistency may be the small sample size of our study. Increasing the sample size may cause a significant difference in the raw REM percentages.

During night sleep, REM sleep has been proposed as a factor aiding corneal oxygenation [16]. The literature shows that extended usage of contact lenses diminishes corneal tissue oxygenation [26]. Our study demonstrated that REM density decreased in OSA patients. However, there is no study to analyze corneal oxygenation data for contact lens usage in OSA patients. Based on this study and a literature review, we believe that OSA patients who wear hard contact lenses might be at risk for corneal thickening. However, this hypothesis requires further studies to be confirmed. Finally, this study is also limited by the lack of a formal control group of individuals who did not have OSA.

To our knowledge, this is the first study showing that OSA patients experience corneal thickness increase during sleep. Because our data are concerned with the acute effect of OSA on the cornea, we only analyzed one night of pre and post sleep data. Therefore, further studies of long term effects of OSA on corneal thickness are required. Our study emphasizes the following points. First, OSA patients who wear hard contact lenses should be monitored carefully, because their corneal thickness can also be affected by decreased REM percentages. Second, it may be prudent for clinicians to consider the possibility of glaucoma in OSA patients and vice versa. Glaucoma is one of the leading causes of visual impairment and blindness [36]. OSA patients have a higher prevalence of glaucoma and ocular hypertension [37], so any patients with increased corneal thickness should be evaluated carefully.

In summary, we do not claim that rapid eye movements are indispensable for a healthy cornea or that the only function of REM sleep is to provide oxygen to the cornea. But our data show that corneal thickness increased in OSA patients, and this finding must be kept in mind when evaluating these patients. Further research would be useful with larger numbers of patients, and longer follow-ups are required to confirm the effect of OSA on the cornea.

## References

[1] Wellman A, White D (2011) Central Sleep Apnea and Periodic Breathing. In: Kryger M, Roth T, WC D, editors. Principles and Practice of Sleep Medicine 5th Edition. Philadelphia, PA: W.B. Saunders Company. pp. 1140–1152.

[2] Buchanan B, Grunstein R (2011) Positive Airway Pressure Treatment for Obstructive Sleep Apnea-Hypopnea Syndrome. In: Kryger M, Roth T, Dement W, editors. Principles and Practice of Sleep Medicine 5th Edition. 5th Edition ed. Philadelphia, PA. pp. 1233–1249.

[3] Aloia MS, Di Dio L, Ilniczky N, Perlis ML, Greenblatt DW, et al (2001) Improving compliance with nasal CPAP and vigilance in older adults with OAHS. Sleep Breath 5: 13–21. 11868136 10.1007/s11325-001-0013-9

[4] Ferini-Strambi L, Baietto C, Di Gioia MR, Castaldi P, Castronovo C, et al (2003) Cognitive dysfunction in patients with obstructive sleep apnea (OSA): partial reversibility after continuous positive airway pressure (CPAP). Brain Res Bull 61: 87–92. 12788211 10.1016/s0361-9230(03)00068-6

[5] Ferini-Strambi L, Marelli S, Galbiati A, Castronovo C (2013) Effects of Continuous Positive Airway Pressure on cognitive function and neuroimaging data in obstructive sleep apnea. Int J Psychophysiol. 10.1016/j.ijpsycho.2013.03.022 23570950

[6] Achermann P, Borbely A (2011) Sleep Homeostasis and Models of Sleep Regulation. In: Kryger M, Roth T, WC D, editors. Principles and Practice of Sleep Medicine 5th Edition. Philadelphia, PA: W.B. Saunders Company. pp. 431–444.

[7] Feng P, Ma Y, Vogel GW (2001) Ontogeny of REM rebound in postnatal rats. Sleep 24: 645–653. 11560177 10.1093/sleep/24.6.645

[8] Feige B, Voderholzer U, Riemann D, Dittmann R, Hohagen F, et al (2002) Fluoxetine and sleep EEG: effects of a single dose, subchronic treatment, and discontinuation in healthy subjects. Neuropsychopharmacology 26: 246–258. 11790520 10.1016/S0893-133X(01)00314-1

[9] Horner RL, Brooks D, Kozar LF, Leung E, Hamrahi H, et al (1998) Sleep architecture in a canine model of obstructive sleep apnea. Sleep 21: 847–858. 9871947

[10] Kleitman N (1963) Sleep and Wakefulness. Chicago: The University of Chicago Press.

[11] Heller H, Glotzbach S, Grahn D (1988) Sleep-Dependent Changes in the Thermoregulatory System. In: Lydic R, Biebuyck J, editors. Clinical Physiology of Sleep. New York: Oxford University Press. pp. 145.

[12] Fisher C, Gorss J, Zuch J (1965) CYCLE OF PENILE ERECTION SYNCHRONOUS WITH DREAMING (REM) SLEEP. PRELIMINARY REPORT. Arch Gen Psychiatry 12: 29–45. 14221689 10.1001/archpsyc.1965.01720310031005

[13] Karacan I (1965) The effect of exciting presleep events on dream reporting and penile erections during sleep. [Doctoral dissertation]. Brooklyn, NY: New York University.

[14] Pessah MA, Roffwarg HP (1972) Spontaneous middle ear muscle activity in man: a rapid eye movement sleep phenomenon. Science 178: 773–776. 4343261 10.1126/science.178.4062.773

[15] Hajak G, Klingelhofer J, Schulz-Varszegi M, Matzander G, Sander D, et al (1994) Relationship between cerebral blood flow velocities and cerebral electrical activity in sleep. Sleep 17: 11–19. 7910702 10.1093/sleep/17.1.11

[16] Maurice DM (1998) The Von Sallmann Lecture 1996: an ophthalmological explanation of REM sleep. Exp Eye Res 66: 139–145. 9533840 10.1006/exer.1997.0444

[17] Hedbys BO, Mishima S (1966) The thickness-hydration relationship of the cornea. Exp Eye Res 5: 221–228. 5914654 10.1016/s0014-4835(66)80010-6

[18] Johnson MH, Boltz RL, Godio LB (1985) Deswelling of the cornea after hypoxia. Am J Optom Physiol Opt 62: 768–773. 4073213 10.1097/00006324-198511000-00008

[19] Klyce SD (1981) Stromal lactate accumulation can account for corneal oedema osmotically following epithelial hypoxia in the rabbit. J Physiol 321: 49–64. 7338822 10.1113/jphysiol.1981.sp013971 PMC1249613

[20] Tai LY, Khaw KW, Ng CM, Subrayan V (2013) Central corneal thickness measurements with different imaging devices and ultrasound pachymetry. Cornea 32: 766–771. 23095499 10.1097/ICO.0b013e318269938d

[21] Kohlhaas M, Boehm AG, Spoerl E, Pursten A, Grein HJ, et al (2006) Effect of central corneal thickness, corneal curvature, and axial length on applanation tonometry. Arch Ophthalmol 124: 471–476. 16606871 10.1001/archopht.124.4.471

[22] Congdon NG, Broman AT, Bandeen-Roche K, Grover D, Quigley HA (2006) Central corneal thickness and corneal hysteresis associated with glaucoma damage. Am J Ophthalmol 141: 868–875. 16527231 10.1016/j.ajo.2005.12.007

[23] Polse KA, Mandell RB (1970) Critical oxygen tension at the corneal surface. Arch Ophthalmol 84: 505–508. 5492460 10.1001/archopht.1970.00990040507021

[24] du Toit R, Vega JA, Fonn D, Simpson T (2003) Diurnal variation of corneal sensitivity and thickness. Cornea 22: 205–209. 12658083 10.1097/00003226-200304000-00004

[25] Moezzi AM, Fonn D, Varikooty J, Richter D (2011) Distribution of overnight corneal swelling across subjects with 4 different silicone hydrogel lenses. Eye Contact Lens 37: 61–65. 21301346 10.1097/ICL.0b013e31820e0bc3

[26] Holden BA, Sweeney DF, Vannas A, Nilsson KT, Efron N (1985) Effects of long-term extended contact lens wear on the human cornea. Invest Ophthalmol Vis Sci 26: 1489–1501. 3863808

[27] Jalbert I, Sweeney DF, Stapleton F (2009) The effect of long-term wear of soft lenses of low and high oxygen transmissibility on the corneal epithelium. Eye (Lond) 23: 1282–1287. 18849917 10.1038/eye.2008.307

[28] Salz JJ, Azen SP, Berstein J, Caroline P, Villasenor RA, et al (1983) Evaluation and comparison of sources of variability in the measurement of corneal thickness with ultrasonic and optical pachymeters. Ophthalmic Surg 14: 750–754. 6646620

[29] Faulkner W, Varley G (1997) Corneal Diagnostic Techniques. In: Krachmer J, Mannis M, Holland E, editors. Cornea Fundamentals of Cornea and External Disease. St Louis, Missouri: Mosby. pp. 279.

[30] Rechtschaffen A, Kales A (1968) A manual of standardized techniques and scoring system for sleep stages in human subjects. Washington D.C.: U.S. Government Printing Office.

[31] Bornstein S (1982) Respiratory monitoring during sleep: polysomnography. In: Guilleminault C, editor. Sleeping and Waking Disorders: Indications and Techniques. Menlo Park, CA.: Addison-Wesley. pp. 183–212.

[32] Bonnet M, Carley D, Guilleminault C (1993) Recording and scoring leg movements. The Atlas Task Force. Sleep 16: 748–759. 8165390

[33] King D, Akiskal HS, Lemmi H, Wilson W, Belluomini J, et al (1981) REM density in the differential diagnosis of psychiatric from medical-neurologic disorders: a replication. Psychiatry Res 5: 267–276. 6948309 10.1016/0165-1781(81)90073-1

[34] Hohagen F, Riemann D, Spiegel R, Holzhauer M, Berger M (1993) Influence of the cholinergic agonist SDZ 210-086 on sleep in healthy subjects. Neuropsychopharmacology 9: 225–232. 8280346 10.1038/npp.1993.58

[35] Antonioli M, Solano L, Torre A, Violani C, Costa M, et al (1981) Independence of REM density from other REM sleep parameters before and after REM deprivation. Sleep 4: 221–225. 7256082 10.1093/sleep/4.2.221

[36] Congdon N, O'Colmain B, Klaver CC, Klein R, Munoz B, et al (2004) Causes and prevalence of visual impairment among adults in the United States. Arch Ophthalmol 122: 477–485. 15078664 10.1001/archopht.122.4.477

[37] Moghimi S, Ahmadraji A, Sotoodeh H, Sadeghniat K, Maghsoudipour M, et al (2013) Retinal nerve fiber thickness is reduced in sleep apnea syndrome. Sleep Med 14: 53–57. 22948081 10.1016/j.sleep.2012.07.004

